# Colonic Lipoma as a Leading Cause of Intussusception Resulting in Bowel Obstruction

**DOI:** 10.7759/cureus.18261

**Published:** 2021-09-24

**Authors:** Meetham Allawati, Tagalsir Logman, Murtadha T Al Qubtan

**Affiliations:** 1 Medicine and Health Sciences, Sultan Qaboos University, Muscat, OMN; 2 General Surgery, Royal Hospital, Muscat, OMN

**Keywords:** case report, intussusception, colonic lipoma, lipoma, bowel obstruction

## Abstract

A colon lipoma is defined as a benign tumor made of adipose tissue present in the submucosa. Lipomas of the colon are unusual with a frequency of 0.035%-4.4%. We present an uncommon case of a 41-year-old female with left colonic lipoma causing intussusception. The patient presented with abdominal pain at the left iliac fossa. She underwent an emergency exploratory laparotomy plus left hemicolectomy and was followed in the surgical out-patient clinic postoperatively. She was found doing well taking a normal diet and having normal bowel habits. The overall prognosis depends on the complete removal of the tumor.

## Introduction

A colon lipoma is defined as a benign tumor made of adipose tissue present in the submucosa [1]. Lipomas of the colon are unusual with a frequency of 0.035%-4.4% [2]. Small lipomas are generally asymptomatic; on the other hand, large lipomas are symptomatic and may result in intussusception and intermittent colonic obstruction. Symptoms include abdominal pain, constipation, lower gastrointestinal bleeding and intussusception [3]. We present an uncommon case of left colonic lipoma causing intussusception.

## Case presentation

A 41-year-old female with no previous comorbidity and no significant medical history presented with a history of abdominal pain at the left iliac fossa (LIF), which was relieved by defecation. She had four previous lower segment cesarian sections (LSCS) the last one being seven years back and an umbilical hernia repair six years back. The patient had constipation for over a few weeks. She felt nauseated and had episodes of vomiting and passed a bloody diarrhea stool. On physical examination, there was tenderness at the LIF with a mild abdominal distention. The patient was stable, appeared to be in pain, afebrile, the abdomen was flatulent, the heart rate was 95/min, and the respiratory rate was 19/min. Laboratory findings were as follows: hemoglobin 13.8 g/dL, hematocrit : 41.3%, platelets 462,000/mm^3^, and white count 7,500/mm^3^.

**Figure 1:**
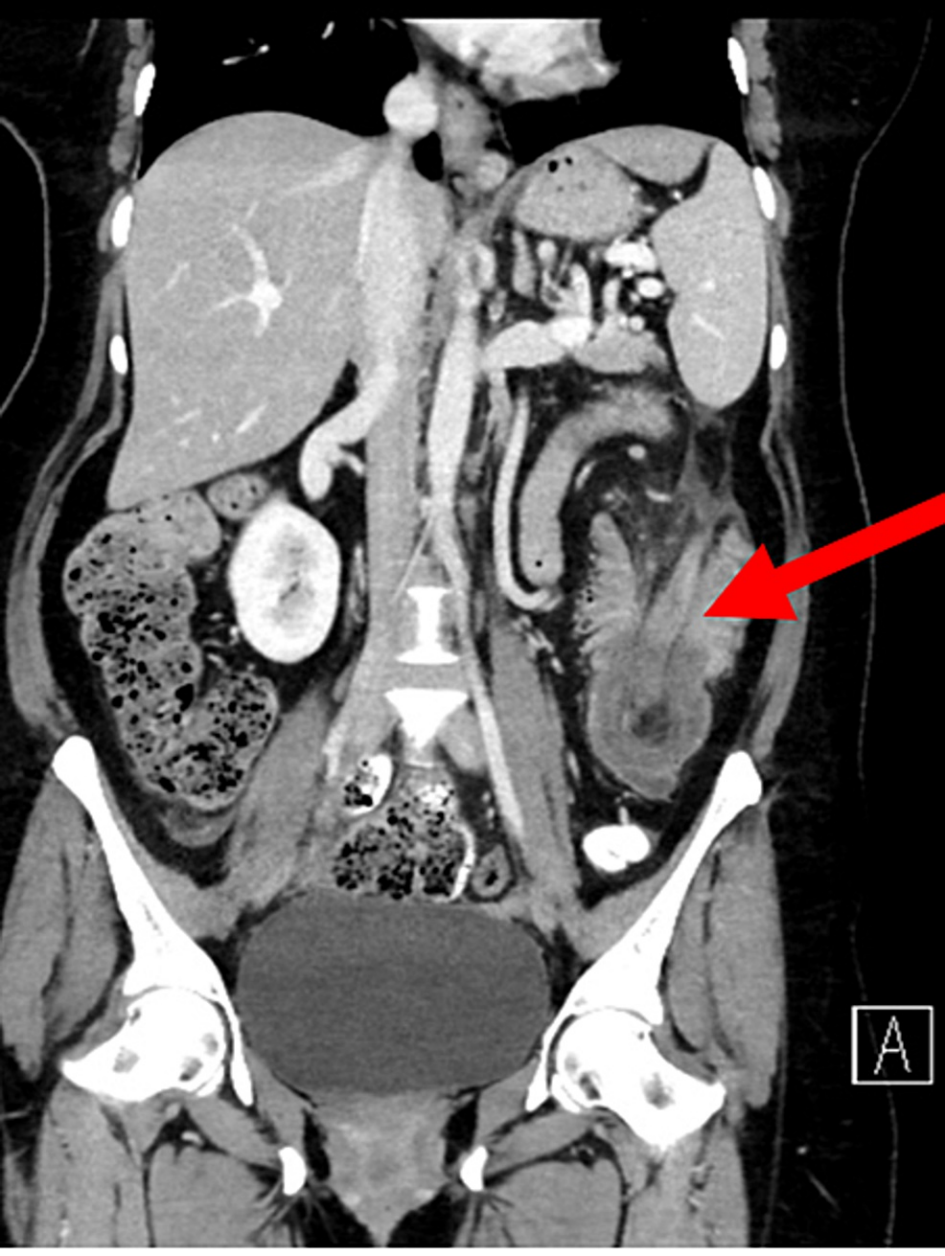
Coronal CT scan showing intussusception

**Figure 2:**
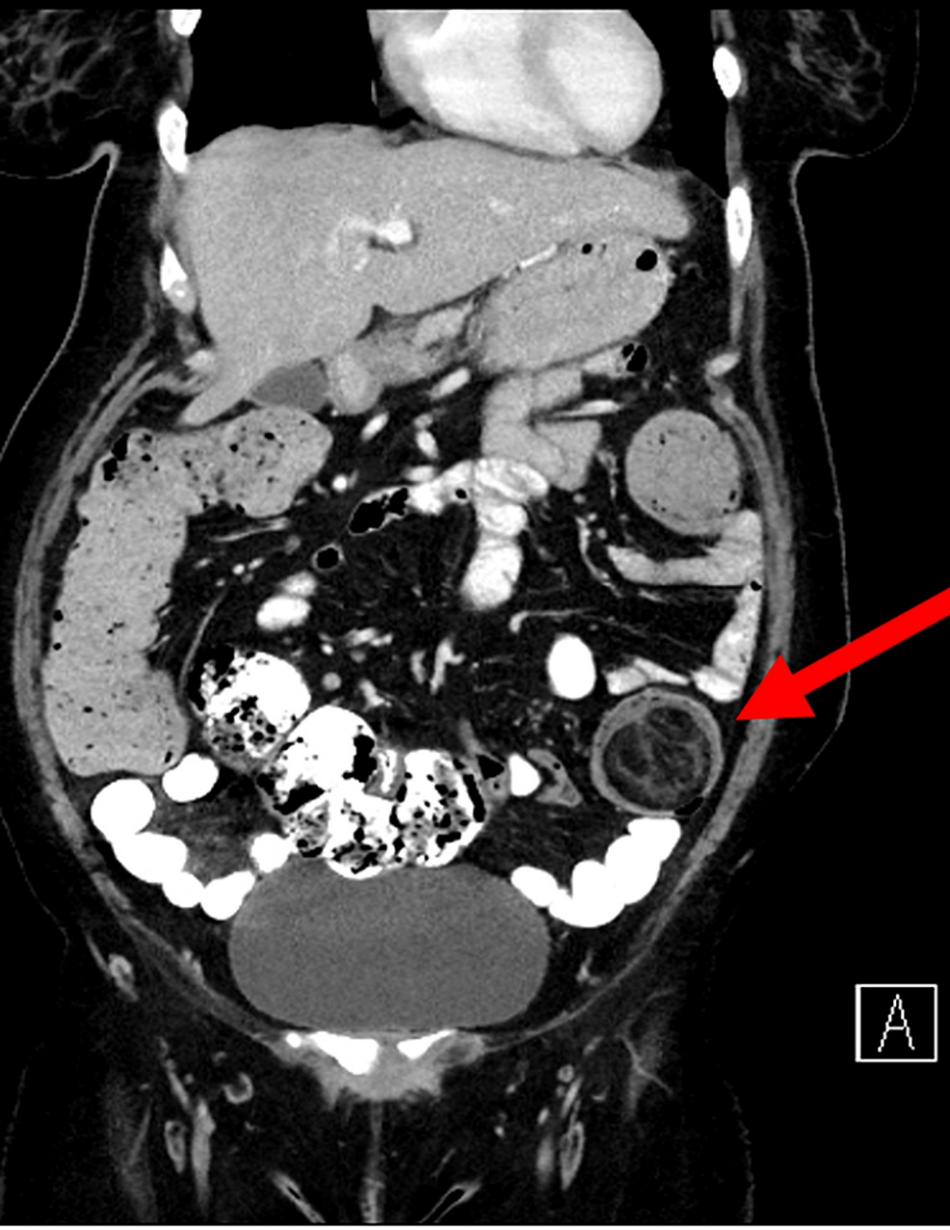
Coronal CT scan showing lipoma

**Figure 3:**
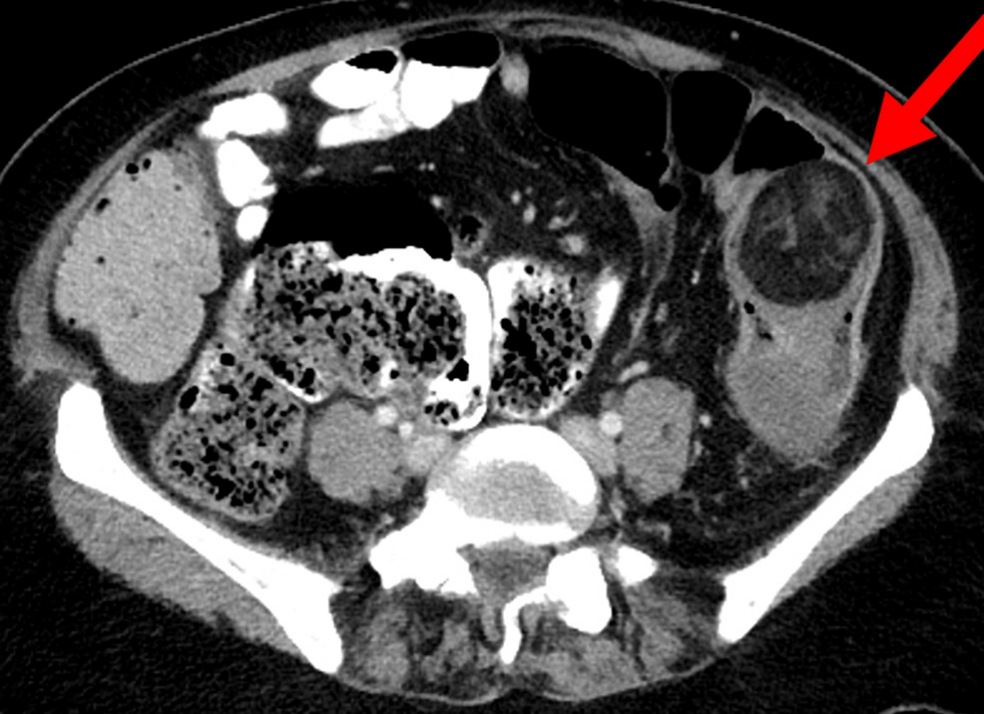
Axial CT scan showing lipoma

A CT abdomen with oral and IV contrast showed an intussusception of the proximal part of the descending colon into its distal part (Figure 1), with a lead of the intussusception in the colonic lipoma measuring 4.6 x 4.2 cm (Figures 2, 3). The length of the intussuscepted segment was about 14 cm. There was a small amount of fluid and gas trapped between the intussusceptum and the intussuscipien. The proximal large bowel loops were distended measuring 5 cm in the transverse colon, and 6.5 cm maximum diameter of the cecum. The small bowel loops were normal in diameter and no evidence of obstruction was seen.

A colonoscopy was done and the finding was a large mass lesion in the descending colon obstructing the lumen and the scope could not be passed beyond the mass. The patient was taken for surgery and underwent exploratory laparotomy and left hemicolectomy. The specimen was sent to the pathology lab and it confirmed a benign lipoma.

The patient stayed few days in the hospital and the post-operative period was uneventful. The histopathology showed an ulcerated lipomatous lesion with hemorrhagic necrosis and perforation. Thirteen reactive lymph nodes were identified with the specimen. The margins were clear. The patient was followed in the surgical out-patient clinic postoperatively and was found doing well taking a normal diet and having normal bowel habits.

## Discussion

Lipomas of the colon were described for the first time by Bauer in 1757 [3,4]. They are relatively rare. Lipomas of the colon are generally asymptomatic, while large ones are associated with complications, so they may require surgery [5]. Most of the patients with intussusception are children, as only 5% of the cases occur in adults [6,7]. Approximately 90% of intussusceptions in adults are caused by an anatomic or pathologic disease. In adults, idiopathic instances without a lead point lesion are uncommon, accounting for 8%-20% of cases. The occurrence of intussusception is low in adults, especially in the descending colon. This is due to the fact that the descending colon has an anatomical attachment to the retroperitoneum [6]. The most frequent sites in the gastrointestinal tract where intussusception occur are the junctions between freely moving segments and the ones fixed by the retroperitoneum or adhesions [7,8]. The exact mechanism of the intussusception of the proximal colon into the distal colon is unknown in adults. It is thought that any lesion within the lumen or in the wall of the bowel that changes the normal peristaltic activity may initiate the intussusception process [7]. In adults, up to 30% of intussusception cases in the small intestine are malignant, while up to 66% of cases of intussusception in the large intestine are malignant [7,9-11]. In most of the adult cases, the treatment of intussusception is surgical, depending on the preoperative diagnosis, size, location of the lipomas, and complications. The majority of authors recommend the excision of the lipomas bigger than 2 cm, especially in the old people, in whom intussusception is more linked to malignancy [12,13]. The prognosis depends on the complete removal of the tumor [13].

## Conclusions

In conclusion, the patient presented with signs of abdominal obstruction. Further investigations including a CT scan showed an intussusception with a posterior abdominal lipoma forming the lead point in this case. The patient was treated surgically, which is the treatment of choice for such conditions. Even though colonic lipomas are rare, they should be put in the differential diagnosis of bowel tumors presenting with symptoms.
